# Mr. Cullen on White Serum

**Published:** 1811-04

**Authors:** P. Cullen

**Affiliations:** Surgeon


*To the Editors of the Medical and Physical Journal.*

GENTLEMEN,

AS you have been pleased to notice, in your last number, my communication to Mr. Brooks of a very rare occurrence; viz. the serum of the blood of the colour and consistence of milk, I thought a few particulars of the case would be also acceptable to you.

The subject of this singular appearance in the blood, is ———— a labourer in the Ordnance department at this place, aged thirty-five, of a thin spare habit of body, and sallow complexion; addicted in the early part of his life to spirituous liquors; latterly to ale or porter.

He first complained of a pain in his left side on the 14th of June, 1810, which, appearing more of a spasmodic than an inflammatory nature, was treated with aperients, fomentations, and volatile liniment; which it yielded to in a few days. Since that, he informs me, he had been subject to loose stools, and transient pains of the same side; but never had recourse to any medicine for these complaints. From his description, the stools must have been of the dysenteric kind. He was not bled for this first attack.

Nov. 24, 1810. In the morning, he came to my house complaining of a return of the pain of his left side. Recollecting the former treatment, I ordered him immediately an aperient draught of the pulv. jalap, comp. the fotus com. and liniment ammon. which he used. In the evening I was sent for to his lodgings. The draught had operated, the fomentation and liniment had been applied. He was rather worse. The pain was very severe, chiefly seated in the middle of the left hypochondrium, extending from that, forward, as far as the cartilago ensiformis, and backward, to the vertebræ. There was little or no tension of the part, but the pain was increased by pressure made along the edges of the costæ spuriæ. He was obliged to bend himself forward to ease the pain, by relaxing the abdominal muscles, and could only lie on his right side, suffering greatly by any attempt made to alter this position. From his first attack in June, he has never been able to lie easy on his left side. He had neither nausea, nor vomiting, nor pain of stomach; no dyspnæa, cough, or any pulmonic affection; and the right hypochondrium was free from any uneasiness. Symptomatic fever had taken place. I could not discover any enlargement of the spleen, but all the symptoms were evidently referred to the region of that viscus. The pulse was rather small, or contracted, quick and frequent; tongue whitish, but moist; skin dry, but not very hot. He was immediately bled to the extent of fourteen or sixteen ounces, and the pulse rose accordingly. The stream of blood was full and *florid*, but on reaching the bason I was surprised to observe a whitish appearance forming, like a cloud, on the surface of the blood, which made me suppose that there had been some milk in in the bason; but I was soon satisfied to the contrary. The bleeding gave relief. He was ordered pulv. antim. with nitre and digitalis in powder, the saline mixture and an anodyne at bed time, the fomentation and liniment to be repeated. Before I left his chamber, I was surprised to see the serum like so much milk, with the crassamentum floating in it.

Nov. 25.—This morning I found the blood as I had left it, the crassamentum floating in serum of the colour and consistence of milk. The crassamentum, on its upper surface, was cupped and sizy, on its under, of a black red colour, and loose in its texture. He took some pills of rhubarb and calomel, and continued the other medicines; and, as the pain was still very severe, he was bled again to the same extent, and with exactly the same appearance. A blister was now ordered. In the evening I was obliged to bleed him the third time, and to the same effect. Each of these successive bleedings exhibited the same phenomena.

Nov. 26.—This day he was so much better as not to require bleeding, and the blister had taken full effect. Continued.

27.—Having some return of pain he was bled the fourth time, to about ten or twelve ounces. The blood had now assumed a very different aspect, the serum was of the natural colour and consistence, the crassamentum was, however, still cupped and sizy, but more florid. Continued.

28.—The pain not being entirely gone he was bled the fifth time, to about eight ounces. The blood was now natural, and the crassamentum neither cupped nor sizy. Contin. med. pro re nata.

After this he continued to mend gradually, and as soon as all the inflammatory symptoms had subsided I put him upon a general course of calomel. He has now returned to duty and seems to be better than before his indisposition. I sent a vial of the serum to Mr. Brooks, of Blenheim Street; and have some still in my possession, which is thicker than it was at first, and emits but very little fœtor.

I here beg leave to correct a mistake in my communication to Mr. Brooks. I there stated "that the splenitis was the consequence of frequent attacks of intermittent fever." But on more particular enquiry I do not find that to be the case. His complaints have been rather of the dysenteric kind, which are, in this place, sometimes attended with a symptomatic fever of the intermittent type. The situation of Sheerness being low and marshy causes intermittents to be endemic here; here; and there are many instances of splenitis, both acute and chronic, the sequelæ of these intermittents.

I am, Gentlemen,
Your most obedient Servant,
P. CULLEN, Surgeon.

